# Thai ethnomedicinal plants as resistant modifying agents for combating *Acinetobacter baumannii* infections

**DOI:** 10.1186/1472-6882-12-56

**Published:** 2012-04-26

**Authors:** Pinanong Na Phatthalung, Sasitorn Chusri, Supayang P Voravuthikunchai

**Affiliations:** 1 Natural Products Research Center and Department of Microbiology, Faculty of Science, Prince of Songkla University, Hat Yai, Songkhla, 90112, Thailand; 2 Faculty of Traditional Thai Medicine, Prince of Songkla University, Hat Yai, Songkhla, 90112, Thailand

## Abstract

**Abstracts:**

**Background**

*Acinetobacter baumannii* is well-recognized as an important nosocomial pathogen, however, due to their intrinsic resistance to several antibiotics, treatment options are limited. Synergistic effects between antibiotics and medicinal plants, particularly their active components, have intensively been studied as alternative approaches.

**Methods**

Fifty-one ethanol extracts obtained from 44 different selected medicinal plant species were tested for resistance modifying agents (RMAs) of novobiocin against *A. baumannii* using growth inhibition assay.

**Results**

At 250 μg/ml, *Holarrhena antidysenterica, Punica granatum, Quisqualis indica, Terminalia bellirica, Terminalia chebula,* and *Terminalia* sp. that possessed low intrinsic antibacterial activity significantly enhanced the activity of novobiocin at 1 μg/ml (1/8xminimum inhibitory concentration) against this pathogen. *Holarrhena antidysenterica* at 7.8 μg/ml demonstrated remarkable resistant modifying ability against *A. baumannii* in combination with novobiocin. The phytochemical study revealed that constituents of this medicinal plant contain alkaloids, condensed tannins, and triterpenoids.

**Conclusion**

The use of *Holarrhena antidysenterica* in combination with novobiocin provides an effective alternative treatment for multidrug resistant *A. baumannii* infections.

## Background

An underestimated nosocomial pathogen, *Acinetobacter baumannii*, is now widely acknowledged as a common bacterium in hospital irrigation and intravenous solutions. It possesses inherent multidrug-resistance (MDR) and the ability to rapidly colonize and infect patients. Moreover, the emergence of acquired MDR by *A. baumannii* to conventional antibiotics presents a serious therapeutic problem in the treatment of the infections [1,2]. Several investigations suggested that synergy effects of plant secondary metabolites and conventional antibiotics could be an alternative way to increase the bacterial susceptibility [3-6].

Plants, particularly ethnomedicinal plants are important sources of natural products. They are rich in a wide variety of secondary metabolites such as tannins, terpenoids, alkaloids, and flavonoids and have been well-established to possess antimicrobial properties [7]. Many plants have been evaluated not only for their inherent antimicrobial activity, but also for their action as a resistant modifying agent (RMA) [4].

Novobiocin, a Gyr B inhibitor, is an effective aminocoumarin drug for the treatment of Gram-positive bacterial infections. However, its low level of activity against Gram-negative pathogens causes a major limitation [8]. Although, several investigations observed synergy and mechanisms of action between natural products and synthetic drugs in effectively combating Gram positive bacterial infections [5], there are a few RMA effective for use with *A. baumannii*[9,10]. Therefore, the aim of this study was to further explore the resistant modifying activity of a wide range of medicinal plants according to their ethnobotanical basis in combination with novobiocin against *A. baumannii*.

## Methods

### Bacterial strain and culture condition

*Acinetobacter baumannii* ATCC 19606 was employed in this study as a model reference strain. The strain was susceptible to ciprofloxacin, colistin, imipenem, and tobramycin and resistant to amikacin, ampicillin, azithromycin, erythromycin, and gentamicin which conducted by disc diffusion method [11]. Well-isolated colonies of *A. baumannii* ATCC 19606 were grown in Mueller Hinton Broth (MHB) (Difco Laboratories, Detroit, MI) at 37°C for 18–24 h. The culture density was adjusted to McFarland standards No. 0.5 and resuspended in MHB to obtain a final concentration of 1 × 10^6^ cfu/ml.

### Medicinal plant materials

Tested medicinal plants are shown in Table 1. Fifty-one ethanol extracts of 44 Thai medicinal plant species were kindly provided by the Natural Products Research Center, Prince of Songkla University, Hat Yai, Thailand [12]. Collected plant materials were washed with distilled water and dried at 60°C overnight. Ground plant material was macerated with 95% ethanol (1:2 w/v) for 7 days. The extract was filtered and evaporated using rotary evaporator at 45°C until it became completely dry. A stock solution (200 mg/ml) was prepared by dissolving 0.2 g of the dried extract in 1 ml of dimethylsulfoxide (DMSO) (Merck, Germany) and stored at −20°C.

**Table 1:** **Intrinsic antibacterial activity and resistant modifying ability of crude extract (250 μg/ml) in combination with novobiocin (1/8xMIC) against*Acinetobacter baumannii*ATCC 19606**

	**Botanical names**	**Family name**	**Part used**	**%Growth inhibition**^**a**^ **± SD**^**b**^		**Interpretation**^**c**^
				**PE**	**PE + NOV**	
1	*Aegle marmelos* (L.) Corr. Serr.	Rutaceae	Fruit	22.10 ± 0.68	27.10 ± 1.38	No synergy
2	*Ardisia colorata* Roxb.	Primulaceae	Fruit	30.17 ± 2.56	39.00 ± 6.09	Synergy
3	*Asclepias curassavica* L.	Asclepiadaceae	Wood	40.81 ± 0.28	43.59 ± 1.78	No synergy
4	*Centella asiatica* (L.) Urb.	Apiaceae	Whole	19.09 ± 1.06	23.93 ± 2.87	No synergy
5	*Cinnamomum bejolghota* (Buch.-Ham.) Sweet	Lauraceae	Wood	58.84 ± 1.37	59.92 ± 1.78	No synergy
			Bark	55.62 ± 4.98	62.44 ± 2.91	No Synergy
6	*Cinnamomum porrectum* (Roxb.) Kosterm.	Lauraceae	Wood	29.72 ± 6.54	26.06 ± 5.21	No synergy
			Bark	56.88 ± 2.14	63.31 ± 4.87	No synergy
7	*Curcuma longa* L.	Zingiberaceae	Rhizome	86.91 ± 2.64	88.78 ± 2.08	No synergy
8	*Curcuma zedoaria* (Christm.) Roscoe	Zingiberaceae	Rhizome	77.73 ± 0.48	79.59 ± 2.62	No synergy
9	*Derris scandens* Benth.	Leguminosea	Stem	49.01 ± 2.37	47.31 ± 3.84	No synergy
10	*Dracaena loureiri* Gagnep.	Agavaceae	Wood	30.08 ± 0.99	29.49 ± 3.19	No synergy
11	*Dryopteris syrmatica* (Willd.) Kuntze	Dryopteridaceae	Stem	17.59 ± 0.41	26.66 ± 5.32	Synergy
12	*Eleutherine americana* (Aubl.) Merr. ex K.	Iridaceae	Bulb	17.87 ± 1.89	22.26 ± 3.12	No synergy
13	*Euphorbia thymifolia* L.	Euphorbiaceae	Whole plant	53.64 ± 0.90	73.99 ± 0.88	Synergy
14	*Garcinia mangostana* L.	Clusiaceae	Pericarp	93.25 ± 3.65	90.48 ± 3.37	No synergy
15	*Gymnopetalum cochinchinensis* (Lour.) Kurz	Cucurbitaceae	Fruit	26.17 ± 0.59	32.45 ± 4.39	No synergy
16	*Holarrhena antidysenterica* (L.) Wall. ex A. DC.	Apocynaceae	Bark	65.88 ± 0.11	94.04 ± 0.59*	Synergy
17	*Impatiens balsamina* L.	Balsaminaceae	Stem	9.77 ± 0.30	12.40 ± 1.56	No synergy
18	*Manilkara achras* (Mill.) Fosb.	Sapotaceae	Fruit	56.59 ± 1.02	63.06 ± 2.97	No synergy
19	*Millingtonia hortensis* L.f.	Bignoniaceae	Flower	28.97 ± 4.30	54.08 ± 0.83	Synergy
20	*Mitragyna speciosa* Korth.	Rubiaceae	Leaf	43.33 ± 2.40	66.15 ± 0.26	Synergy
21	*Momordica charantia* L.	Cucurbitaceae	Vine	22.26 ± 0.85	25.79 ± 3.10	No synergy
22	*Morinda citrifolia* L.	Rubiaceae	Fruit	16.96 ± 0.63	25.86 ± 1.22	Synergy
23	*Murdannia loriformis* (Hassk.) R. Rao & Kammathy	Commilinaceae	Whole plant	16.42 ± 1.51	22.04 ± 1.67	No synergy
24	*Oroxylum indicum* (L.) Vent.	Bignoniaceae	Leaf	67.18 ± 1.59	71.30 ± 5.28	No synergy
25	*Peltophorum pterocarpum* (DC.) Backer ex K. Heyne	Fabaceae	Flower	42.80 ± 0.43	47.83 ± 4.49	No synergy
			Bark	78.26 ± 0.60	88.75 ± 6.10	Synergy
26	*Piper betle* L.	Piperaceae	Leaf	42.72 ± 0.13	39.92 ± 3.43	No synergy
27	*Piper nigrum* L.	Piperaceae	Fruit	38.07 ± 1.96	42.24 ± 2.60	No synergy
			Seed	29.07 ± 0.75	31.47 ± 3.27	No synergy
28	*Piper retrofractum* Vahl	Piperaceae	Fruit	44.02 ± 1.08	49.80 ± 4.19	No synergy
29	*Piper sarmentosum* Roxb	Piperaceae	Leaf	20.70 ± 0.88	25.02 ± 0.62	No synergy
30	*Pluchea indica* (L.) Less.	Asteraceae	Leaf	26.64 ± 0.97	53.59 ± 3.60*	Synergy
31	*Psidium guajava* L.	Myrtaceae	Leaf	71.24 ± 2.00	81.19 ± 1.50*	Synergy
32	*Punica granatum* L.	Puniceaceae	Pericarp	72.58 ± 1.20	99.29 ± 0.63*	Synergy
33	*Quercus infectoria* G.Olivier	Fagaceae	Gall	89.09 ± 0.15	88.77 ± 1.00	No synergy
34	*Quisqualis indica* L.	Combretaceae	Flower	79.22 ± 0.28	94.63 ± 2.62*	Synergy
35	*Rhizophora mucronata* Lam.	Rhizophoraceae	Fruit	44.64 ± 0.59	53.35 ± 2.56	Synergy
			Bark	42.68 ± 8.20	53.03 ± 4.95	Synergy
36	*Rhodomyrtus tomentosa* (Aiton) Hassk.	Myrtaceae	Stem	77.01 ± 1.28	81.81 ± 4.01	No synergy
37	*Sandoricum indicum* Cav.	Meliaceae	Root	65.24 ± 1.32	66.94 ± 2.13	No synergy
38	*Tamarindus indica* L.	Fabaceae	Leaf	19.76 ± 1.55	25.03 ± 3.45	No synergy
39	*Terminalia bellirica* (Gaertn.) Roxb.	Combretaceae	Fruit	74.79 ± 0.53	95.68 ± 1.14*	Synergy
40	*Terminalia chebula* (Gaertn.) Retz.	Combretaceae	Fruit	61.25 ± 0.42	94.33 ± 1.95*	Synergy
41	*Terminalia* sp.	Combretaceae	Fruit	79.53 ± 0.24	95.92 ± 1.10*	Synergy
42	*Theobroma cacao* L.	Sterculiaceae	Pericarp	17.35 ± 0.74	22.81 ± 0.68	No synergy
			Seed	19.25 ± 1.08	29.61 ± 4.13	Synergy
43	*Vitex trifolia* L.	Verbenaceae	Leaf	22.12 ± 0.68	28.65 ± 3.57	No synergy
44	*Xylocarpus granatum* J. Koenig.	Meliaceae	Pericarp	52.39 ± 3.48	53.27 ± 1.91	No synergy
			Seed	44.27 ± 5.13	54.55 ± 3.66	No synergy

Percentage of growth inhibition of novobiocin against *A. buamannii* ATCC 19606 was 6.67%.

^a^Percentage of growth inhibition in the present of plant extract (PE) and plant extract in combination with novobiocin (PE + NOV) against *A. buamannii* ATCC 19606.

^b^SD Standard Deviation.

^c^Synergy: (PE + NOV) > (PE) + (NOV); No synergy: (PE + NOV) < (PE) + (NOV) [6].

*P < 0.01: Significantly different from the effect of plant extract.

### Determination of minimum inhibitory concentration (MIC) of novobiocin

The MIC of novobiocin was determined by the broth microdilution method as described by the Clinical and Laboratory Standard Institute (CLSI) [13].

### Intrinsic antibacterial activity and resistant modifying ability of medicinal plant extracts

Intrinsic antibacterial activities were determined by growth inhibition assays [9]. The bacterial culture (100 μl) was inoculated into a 96-well microtiter plate containing 50 μl of crude extracts (1,000 μg/ml) and 50 μl of MHB and then incubated at 37°C for 18 h. The intrinsic antibacterial activity was exhibited as the percentage of growth inhibition and calculated from the following equation:

(1) $$\%\text{Growth inhibition}=\left[\left({\text{OD}}_{\text{A}}–{\text{OD}}_{\text{B}}\right)\times 100\right]/{\text{OD}}_{\text{A}}$$

Where OD_A_ is Optical density (OD) 595 nm of bacteria culture in MHB supplemented with 1%DMSO as positive control and OD_B_ is OD 595 nm of the bacterial culture in MHB supplemented with plant extracts.

Resistant modifying ability of the extracts was observed by adding of 50 μl novobiocin at a concentration of 1/8xMIC (1 μg/ml) into the tested plate instead of MHB. This biological activity was exhibited as the percentage of growth inhibition as well but calculated from the following equation, where OD_C_ is OD 595 nm of the bacterial culture in MHB supplemented with the plant extract in combination with novobiocin:

(2) $$\%\text{Growth inhibition}=\left[\left({\text{OD}}_{\text{A}}–{\text{OD}}_{\text{C}}\right)\times 100\right]/{\text{OD}}_{\text{A}}$$

Effective medicinal plants that demonstrated a synergistic effect with novobiocin and exhibited bacterial growth inhibition more than 90% were selected for further experiments. The efficacy of combination therapy of the promising medicinal plants with novobiocin was additionally determined by measuring the resistant modifying capabilities of the extracts at varying concentrations ranging from 7.8 to 250 μg/ml.

### Phytochemical screening methods

Phytochemical screening tests for alkaloids, condensed tannins, flavonoids, hydrolysable tannins, steroids, and triterpenes were qualitatively analyzed by standard colour tests as previously described [14].

## Results and discussion

Intrinsic resistance of *A. baumannii* to novobiocin was observed with MIC value at 8 μg/ml. As shown in Table 1, 48 out of 51 tested ethanol extracts at concentration of 250 μg/ml had low inherent antibacterial activity (% of bacterial growth inhibition was less than 80%). In combination with the antibiotic, the extracts of 18 medicinal plants demonstrated synergistic interaction against *A. baumannii*. Interestingly, the bacterial growth inhibition in the presence of novobiocin in combination with the extracts of *Holarrhena antidysenterica, Punica granatum, Quisqualis indica, Terminalia bellirica, Terminalia chebula,* and *Terminalia* sp. extracts was significantly higher than the intrinsic antibacterial activity of the extracts (Table 1).

**Figure 1:**
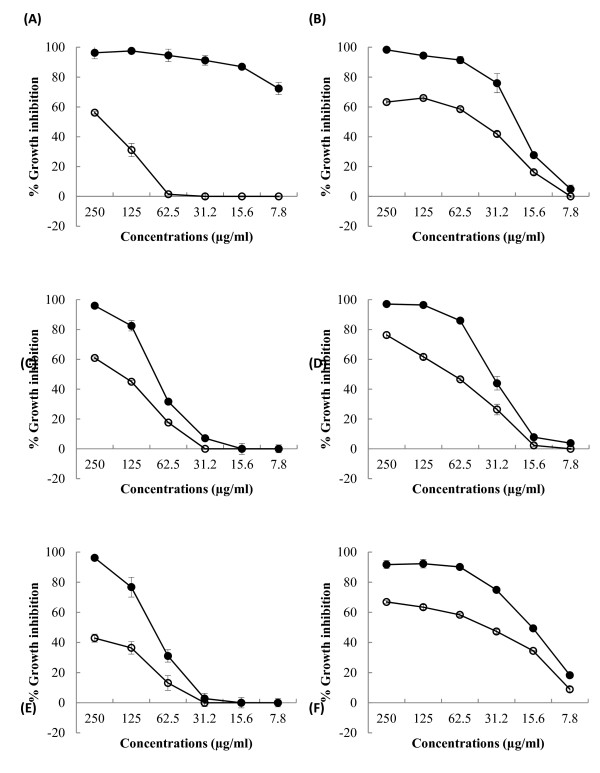
**Bacterial growth inhibition of*Holarrhena antidysenterica*(A),*Punica granatum*(B),*Quisqualis indica*(C),*Terminalia bellirica*(D),*Terminalia chebula*(E), and*Terminalia*sp. (F) ethanol extracts (○) and the extracts in combination with 1/8xMIC of novobiocin (●) against*Acinetobacter baumannii*ATCC 19606.** Percentage of bacterial growth inhibition of 1/8xMIC of novobiocin on this pathogen was 6.67%.

To explore the potential of developing a more powerful combination therapy of these medicinal plants with novobiocin, we determined the resistant modifying ability of varying concentrations of the extracts from 7.8 to 250 μg/ml by growth inhibition assay as illustrated in Figure 1. *Holarrhena antidysenterica* extract which concentrations ranging from 7.8 to 62.5 μg/ml possessed no intrinsic anti-acinetobacter activity (Figure 1A) was demonstrated to be a powerful RMA in combination with novobiocin against this pathogen.

Our preliminary phytochemical test revealed that alkaloids were common principles among the effective extracts. In addition to alkaloids, other compounds including condensed tannins, triterpenoids, flavonoids, hydrolysable tannins, and steroids were detected (Table 2). Although the antibiotic resistant modifying ability of active principles of the effective medicinal plants has never been investigated, plant-derived alkaloids have been well-clarified as efflux pump inhibitors (EPIs) for Gram positive bacteria [15,16]. Recent evaluation of 13 phyto-alkaloids for their EPI potential against staphylococcal isolates revealed that 60% and 30% of the tested compounds exhibited the activity against methicillin resistant *Staphylococcus aureus* (MRSA) and methicillin susceptible *S. aureus* (MSSA), respectively [16]. Four plant-derived alkaloids consisting of reserpine, quinine, harmaline, and piperine possessed notable potential EPI activities on both MRSA and MSSA [16]. More importantly, their mechanisms of actions as a RMA have been proposed. Piperine was recorded as an inhibitor of MdeA [17] and NorA [18] efflux pumps of *S. aureus* and Rv1258c efflux pump of *Mycobacterium tuberculosis*[19]. Reserpine was found as an inhibitor of Bmr efflux pump in *Bacillus subtilis*, Tet(K) and NorA efflux pumps of *S. aureus*[20]. In addition to phyto-alkaloids, several plant-derived polyphenols such as epigallocatechin gallate of *Camellia sinesis*, tellimagrandin I and rugosin B isolated from *Rosa canina* have been established as useful RMAs with different mechanisms of actions including inhibitions of adapted drug target sites or enzymatic degradation of drugs [4]. Intensive investigations on plant-derived compounds as RMAs have been performed in Gram-positive, but relatively very few studies have been carried out to evaluated RMA activities of plant-derived compounds on Gram-negative bacteria [21-23].

**Table 2:** Extraction yields and phytochemical constituents of tested medicinal plant extracts

	**Botanical names**	**Part used**	**Yield (%; w/w)**^**a**^	**Phytochemical constituents**^**b**^					
				**1**	**2**	**3**	**4**	**5**	**6**
1	*Aegle marmelos* (L.) Corr. Serr.	Fruit	5.3	+	+	+	-	+	-
2	*Ardisia colorata* Roxb.	Fruit	4.4	+	+	-	-	+	-
3	*Asclepias curassavica* L.	Wood	0.9	+	+	-	-	-	-
4	*Centella asiatica* (L.) Urb.	Whole	6.0	+	-	-	-	+	-
5	*Cinnamomum bejolghota* (Buch.-Ham.) Sweet	Wood	2.2	+	+	-	-	+	-
		Bark	14.6	+	-	-	+	+	-
6	*Cinnamomum porrectum* (Roxb.) Kosterm.	Wood	11.2	-	-	-	-	+	-
		Bark	7.0	+	+	-	-	+	-
7	*Curcuma longa* L.	Rhizome	13.9	+	+	+	-	+	-
8	*Curcuma zedoaria* (Christm.) Roscoe	Rhizome	13.9	+	+	+	-	-	+
9	*Derris scandens* Benth.	Stem	3.2	-	+	-	-	+	-
10	*Dracaena loureiri* Gagnep.	Wood	16.9	-	-	-	-	-	+
11	*Dryopteris syrmatica* (Willd.) Kuntze	Stem	4.5	+	+	-	-	+	-
12	*Eleutherine americana* (Aubl.) Merr. ex K.	Bulb	4.8	+	+	-	-	-	-
13	*Euphorbia thymifolia* L.	Whole plant	1.3	-	+	-	-	+	-
14	*Garcinia mangostana* L.	Pericarp	5.3	-	-	-	-	-	-
15	*Gymnopetalum cochinchinensis* (Lour.) Kurz	Fruit	7.6	-	-	-	-	+	-
16	*Holarrhena antidysenterica* (L.) Wall. ex A. DC.	Bark	2.1	+	+	-	-	-	+
17	*Impatiens balsamina* L.	Stem	5.2	-	+	-	-	+	-
18	*Manilkara achras* (Mill.) Fosb.	Fruit	26.7	+	-	+	-	-	+
19	*Millingtonia hortensis* L.f.	Flower	25.4	+	+	+	-	-	-
20	*Mitragyna speciosa* Korth.	Leaf	5.9	+	+	-	-	+	-
21	*Momordica charantia* L.	Vine	3.0	+	-	-	-	+	-
22	*Morinda citrifolia* L.	Fruit	7.3	+	-	+	-	+	-
23	*Murdannia loriformis* (Hassk.) R. Rao & Kammathy	Whole plant	7.6	+	-	-	-	+	-
24	*Oroxylum indicum* (L.) Vent.	Leaf	3.7	+	+	-	-	+	-
25	*Peltophorum pterocarpum* (DC.) Backer ex K. Heyne	Flower	7.1	+	-	-	-	-	-
		Bark	7.1	+	+	-	-	-	+
26	*Piper betle* L.	Leaf	12.4	-	+	-	-	+	-
27	*Piper nigrum* L.	Fruit	4.2	+	-	-	-	+	-
		Seed	4.2	+	-	-	-	+	-
28	*Piper retrofractum* Vahl	Fruit	7.0	-	-	-	-	+	-
29	*Piper sarmentosum* Roxb	Leaf	1.7	+	-	-	-	+	-
30	*Pluchea indica* (L.) Less.	Leaf	17.8	+	+	-	-	+	-
31	*Psidium guajava* L.	Leaf	8.0	+	+	-	-	+	-
32	*Punica granatum* L.	Pericarp	13.0	+	+	+	-	-	+
33	*Quercus infectoria* G.Olivier	Gall	37.8	+	-	-	+	-	-
34	*Quisqualis indica* L.	Flower	11.0	+	-	+	+	+	-
35	*Rhizophora mucronata* Lam.	Fruit	10.7	+	+	-	-	-	+
		Bark	11.6	-	+	-	-	-	+
36	*Rhodomyrtus tomentosa* (Aiton) Hassk.	Stem	7.1	+	+	-	-	-	+
37	*Sandoricum indicum* Cav.	Root	4.0	+	-	-	-	+	-
38	*Tamarindus indica* L.	Leaf	4.8	+	+	+	-	+	-
39	*Terminalia bellirica* (Gaertn.) Roxb.	Fruit	14.8	+	-	-	-	+	-
40	*Terminalia chebula* (Gaertn.) Retz.	Fruit	5.9	+	+	-	-	-	+
41	*Terminalia* sp.	Fruit	23.9	+	-	-	+	-	-
42	*Theobroma cacao* L.	Pericarp	3.6	+	+	-	-	+	-
		Seed	5.9	-	+	+	-	-	+
43	*Vitex trifolia* L.	Leaf	ND^c^	+	+	-	-	+	-
44	*Xylocarpus granatum* J. Koenig.	Pericarp	2.6	+	+	-	-	+	-
		Seed	6.7	+	+	+	-	-	+

^a^Percentage extract yields of medicinal plants were weight of crude extract per 100 g of dried plant materials.

^b^Phytochemincal constituents: 1, alkaloids; 2, condensed tannins; 3, flavonoids; 4, hydrolysable tannins; 5, steroids and 6, triterpenoids; ‘-’ indicates absence of phytoconstituents ‘+’ indicates presence of phytoconstituents.

^c^*ND* Not determined.

In the last decade multidrug resistance in *A. baumannii* became a serious growing problem worldwide. Colistin, an old antibiotic with risk toxicity, has recently been brought back into use to treat MDR bacteria as a stopgap measure until new antibiotics can be developed [24]. A number of workers have proposed the synergistically action combination of conventional antibiotics with RMA act synergistically against MDR Gram-negative bacteria [4,25,26]. We have demonstrated that certain plant ethanol extracts significantly enhanced the activity of novobiocin against *A. baumannii*. *Holarrhena antidysenterica* is of interest since the extract at 7.8 to 62.5 μg/ml possessed no intrinsic antibacterial activity, but in combination with sub-MIC of novobiocin led to a marked decrease in the bacterial growth. Alkaloids were proposed as active principles of the plant that possessed antibacterial activity on *S. aureusS. epidermidisStreptococcus faecalisB. subtilisEscherichia coli*, and *Pseudomonas aeruginosa*[27-29]. Some of the alkaloids such as pubadysone, pubescine, norholadiene, pubescimine, puboestrene, pubamide, and naringenin was isolated form bark, seeds, and leaves of this plant [30-32].

Our previous investigation demonstrated that ellagic acid which acts as an efflux pump inhibitor exhibited a synergistic effect with novobiocin and other aminocoumarins against both *A. baumannii* ATCC 19606 and MDR *A. baumannii*[9]. Ethylenediaminetetraacetic acid and polyethyleneimine that disturb outer membrane permeability have been reported as RMA for novobiocin against *P. aeruginosa* and *Stenotrophomonas morelense*[33,34]. Similarly, berry-derived phenolic compounds that efficiently destabilized outer membrane permeability resulted in increase in novobiocin susceptibility of *Salmonella enterica* serotype Typhimurium [35].

Since intrinsic novobiocin resistance in *A. baumannii* is related to the synergistic interaction between limited outer membrane permeability and energy-dependent multidrug efflux pumps [36,37], the RMA for novobiocin possibly acts as a permeabilizer and/or an efflux pump inhibitor.

## Conclusion

The RMA activity of Thai medicinal plants in combination with novobiocin against *A. baumannii* is reported for the first time. These findings led us to the development of a new generation of phytopharmaceuticals that using plant-derived compounds in combination with existing antibiotics to treat MDR *A. baumannii* that currently are almost untreatable. Its mechanism of action as well as the active constituents of a promising plant, *Holarrhena antidysenterica* should be further investigated.

## Competing interests

The authors declare that they have no competing interests.

## Author’ contributions

PN designed and carried out the study. SC and SV supervised in the design of the study and contributed to the writing process. All authors read and approved the final manuscript.

## Pre-publication history

The pre-publication history for this paper can be accessed here:

http://www.biomedcentral.com/1472-6882/12/56/prepub

## References

[B1] Acosta J Merino M Viedma E Poza M Sanz F Otero JR Chaves F Bou G Multidrug-resistant Acinetobacter baumannii Harboring OXA-24 Carbapenemase, Spain Emerg Infect Dis 2011 17 1064 1067 21749771 10.3201/eid1706.091866 PMC3358182

[B2] Katsaragakis S Markogiannakis H Samara E Pachylaki N Theodoraki EM Xanthaki A Toutouza M Toutouzas KG Theodorou D Predictors of mortality of Acinetobacter baumannii infections: A 2-year prospective study in a Greek surgical intensive care unit Am J Infect Control 2010 38 631 635 20471716 10.1016/j.ajic.2010.01.009

[B3] Hemaiswarya S Doble M Synergistic interaction of eugenol with antibiotics against Gram negative bacteria Phytomedicine 2009 16 997 1005 19540744 10.1016/j.phymed.2009.04.006

[B4] Hemaiswarya S Kruthiventi AK Doble M Synergism between natural products and antibiotics against infectious diseases Phytomedicine 2008 15 639 652 18599280 10.1016/j.phymed.2008.06.008

[B5] Ulrich-Merzenich G Panek D Zeitler H Vetter H Wagner H Drug development from natural products: exploiting synergistic effects Indian J Exp Biol 2010 48 208 219 21046973

[B6] Wagner H Ulrich-Merzenich G Synergy research: approaching a new generation of phytopharmaceuticals Phytomedicine 2009 16 97 110 19211237 10.1016/j.phymed.2008.12.018

[B7] Perumal Samy R Gopalakrishnakone P Therapeutic potential of plants as aAnti-microbials for drug discovery Evid Based Complement Alternat Med 2010 7 283 294 18955349 10.1093/ecam/nen036 PMC2887332

[B8] Kampranis SC Maxwell A Conversion of DNA gyrase into a conventional type II topoisomerase Proc Natl Acad Sci U S A 1996 93 14416 14421 8962066 10.1073/pnas.93.25.14416 PMC26147

[B9] Chusri S Villanueva I Voravuthikunchai SP Davies J Enhancing antibiotic activity: a strategy to control Acinetobacter infections J Antimicrob Chemother 2009 64 1203 1211 19861335 10.1093/jac/dkp381

[B10] Rosato A Piarulli M Corbo F Muraglia M Carone A Vitali ME Vitali C In vitro synergistic antibacterial action of certain combinations of gentamicin and essential oils Curr Med Chem 2010 17 3289 3295 20666717 10.2174/092986710792231996

[B11] Clinical and Laboratory Standards Institute M02-A10-Performance Standards for Antimicrobial Disk Susceptibility Tests Approved Standard-Tenth Edition 2009 Clinical and Laboratory Standards Institute Wayne, Pennsylvania, USA

[B12] Voravuthikunchai SP Limsuwan S Chusri S Govil GN, Singh VK New Perspectives on Herbal Medicines for Treating Bacterial Infections Recent Progress in Medicinal Plants: Chronic and Common Diseases-IV 2006 Studium Press, Houstan, Texas, USA 41 101

[B13] Clinical and Laboratory Standards Institute M07-A8-Methods for Dilution Antimicrobial Susceptibility Tests for Bacteria That Grow Aerobically Approved Standard-Eighth Edition 2009 Clinical and Laboratory Standards Institute Wayne, Pennsylvania, USA

[B14] Kaur GJ Arora DS Antibacterial and phytochemical screening of Anethum graveolens, Foeniculum vulgare and Trachyspermum ammi BMC Complement Altern Med 2009 9 30 19656417 10.1186/1472-6882-9-30 PMC2736926

[B15] Kamicker BJ Sweeney MT Kaczmarek F Dib-Hajj F Shang W Crimin K Duignan J Gootz TD Bacterial efflux pump inhibitors Methods Mol Med 2008 142 187 204 18437315 10.1007/978-1-59745-246-5_15

[B16] Mohtar M Johari SA Li AR Isa MM Mustafa S Ali AM Basri DF Inhibitory and resistance-modifying potential of plant-based alkaloids against methicillin-resistant Staphylococcus aureus (MRSA) Curr Microbiol 2009 59 181 186 19475447 10.1007/s00284-009-9416-9

[B17] Mirza ZM Kumar A Kalia NP Zargar A Khan IA Piperine as an inhibitor of the MdeA efflux pump of Staphylococcus aureus J Med Microbiol 2011 60 1472 1478 21680766 10.1099/jmm.0.033167-0

[B18] Nargotra A Sharma S Koul JL Sangwan PL Khan IA Kumar A Taneja SC Koul S Quantitative structure activity relationship (QSAR) of piperine analogs for bacterial NorA efflux pump inhibitors Eur J Med Chem 2009 44 4128 4135 19523722 10.1016/j.ejmech.2009.05.004

[B19] Sharma S Kumar M Sharma S Nargotra A Koul S Khan IA Piperine as an inhibitor of Rv1258c, a putative multidrug efflux pump of Mycobacterium tuberculosis J Antimicrob Chemother 2010 65 1694 1701 20525733 10.1093/jac/dkq186

[B20] Stavri M Piddock LJV Gibbons S Bacterial efflux pump inhibitors from natural sources J Antimicrob Chemother 2007 59 1247 1260 17145734 10.1093/jac/dkl460

[B21] Darwish RM Aburjai TA Effect of ethnomedicinal plants used in folklore medicine in Jordan as antibiotic resistant inhibitors on Escherichia coli BMC Complement Altern Med 2010 10 10.1186/1472-6882-10-9 PMC2839964 20187978

[B22] Fadli M Saad A Sayadi S Chevalier J Mezrioui NE Pagès JM Hassani L Antibacterial activity of Thymus maroccanus and Thymus broussonetii essential oils against nosocomial infection - bacteria and their synergistic potential with antibiotics Phytomedicine 2012 15 464 471 22257599 10.1016/j.phymed.2011.12.003

[B23] Fadli M Chevalier J Saad A Mezrioui NE Hassani L Pages JM Essential oils from Moroccan plants as potential chemosensitisers restoring antibiotic activity in resistant Gram-negative bacteria Int J Antimicrob Agents 2011 38 325 330 21752605 10.1016/j.ijantimicag.2011.05.005

[B24] Zavascki AP Goldani LZ Li J Nation RL Polymyxin B for the treatment of multidrug-resistant pathogens: a critical review J Antimicrob Chemother 2007 60 1206 1215 17878146 10.1093/jac/dkm357

[B25] Coban AY Tanriverdi Cayci Y Erturan Z Durupinar B Effects of efflux pump inhibitors phenyl-arginine-beta-naphthylamide and 1-(1-naphthylmethyl)-piperazine on the antimicrobial susceptibility of Pseudomonas aeruginosa isolates from cystic fibrosis patients J Chemother 2009 21 592 594 19933053 10.1179/joc.2009.21.5.592

[B26] Peleg AY Adams J Paterson DL Tigecycline efflux as a mechanism for nonsusceptibility in Acinetobacter baumannii Antimicrob Agents Chemother 2007 51 2065 2069 17420217 10.1128/AAC.01198-06 PMC1891386

[B27] Chakraborty A Brantner AH Antibacterial steroid alkaloids from the stem bark of Holarrhena pubescens J Ethnopharmacol 1999 15 339 344 10624899 10.1016/s0378-8741(99)00119-1

[B28] Kavitha D Shilpa PN Devaraj SN Antibacterial and antidiarrhoeal effects of alkaloids of Holarrhena antidysenterica WALL Indian J Exp Biol 2004 42 589 594 15260110

[B29] Kavitha D Niranjali S Inhibition of enteropathogenic Escherichia coli adhesion on host epithelial cells by Holarrhena antidysenterica (L.) WALL Phytother Res 2009 23 1229 1236 19441013 10.1002/ptr.2520

[B30] Kumar A Ali M A new steroidal alkaloid from the seeds of Holarrhena antidysenterica Fitoterapia 2000 71 101 104 10727803 10.1016/s0367-326x(99)00111-2

[B31] Siddiqui BS Usmani SB Ali ST Begum S Rizwani GH Further constituents from the bark of Holarrhena pubescens Phytochemistry 2001 58 1199 1204 11738407 10.1016/s0031-9422(01)00330-2

[B32] Tuntiwachwuttikul P Pootaeng-on Y Phansa P Limpachayaporn P Charoenchai P Taylor WC Constituents of the leaves of Holarrhena pubescens Fitoterapia 2007 78 271 273 17329039 10.1016/j.fitote.2006.11.003

[B33] Alakomi HL Paananen A Suihko ML Helander IM Saarela M Weakening effect of cell permeabilizers on gram-negative bacteria causing biodeterioration Appl Environ Microbiol 2006 72 4695 4703 16820461 10.1128/AEM.00142-06 PMC1489302

[B34] Khalil H Chen T Riffon R Wang R Wang Z Synergy between polyethylenimine and different families of antibiotics against a resistant clinical isolate of Pseudomonas aeruginosa Antimicrob Agents Chemother 2008 52 1635 1641 18285485 10.1128/AAC.01071-07 PMC2346620

[B35] Alakomi HL Puupponen-Pimia R Aura AM Helander IM Nohynek L Oksman-Caldentey KM Saarela M Weakening of salmonella with selected microbial metabolites of berry-derived phenolic compounds and organic acids J Agric Food Chem 2007 55 3905 3912 17439151 10.1021/jf070190y

[B36] Damier-Piolle L Magnet S Bremont S Lambert T Courvalin P AdeIJK, a resistance-nodulation-cell division pump effluxing multiple antibiotics in Acinetobacter baumannii Antimicrob Agents Chemother 2008 52 557 562 18086852 10.1128/AAC.00732-07 PMC2224764

[B37] Savage PB Multidrug-resistant bacteria: overcoming antibiotic permeability barriers of gram-negative bacteria Ann Med 2001 33 167 171 11370769 10.3109/07853890109002073

